# Traditional knowledge of wild edible plants used in Palestine (Northern West Bank): A comparative study

**DOI:** 10.1186/1746-4269-4-13

**Published:** 2008-05-12

**Authors:** Mohammed S Ali-Shtayeh, Rana M Jamous, Jehan H Al-Shafie', Wafa' A Elgharabah, Fatemah A Kherfan, Kifayeh H Qarariah, Isra' S Khdair, Israa M Soos, Aseel A Musleh, Buthainah A Isa, Hanan M Herzallah, Rasha B Khlaif, Samiah M Aiash, Ghadah M Swaiti, Muna A Abuzahra, Maha M Haj-Ali, Nehaya A Saifi, Hebah K Azem, Hanadi A Nasrallah

**Affiliations:** 1 Department of Biology and Biotechnology, An-Najah University, Nablus, Palestine; 2 Biodiversity and Biotechnology Research Unit, Biodiversity and Environmental Research Center, BERC, Til, Nablus, Palestine

## Abstract

**Background:**

A comparative food ethnobotanical study was carried out in fifteen local communities distributed in five districts in the Palestinian Authority, PA (northern West Bank), six of which were located in Nablus, two in Jenin, two in Salfit, three in Qalqilia, and two in Tulkarm. These are among the areas in the PA whose rural inhabitants primarily subsisted on agriculture and therefore still preserve the traditional knowledge on wild edible plants.

**Methods:**

Data on the use of wild edible plants were collected for one-year period, through informed consent semi-structured interviews with 190 local informants. A semi-quantitative approach was used to document use diversity, and relative importance of each species.

**Results and discussion:**

The study recorded 100 wild edible plant species, seventy six of which were mentioned by three informants and above and were distributed across 70 genera and 26 families. The most significant species include *Majorana syriaca, Foeniculum vulgare, Malvasylvestris*, *Salvia fruticosa, Cyclamen persicum, Micromeria fruticosa, Arum palaestinum, Trigonella foenum-graecum*, *Gundelia tournefortii*, and *Matricaria aurea*. All the ten species with the highest mean cultural importance values (mCI), were cited in all five areas. Moreover, most were important in every region. A common cultural background may explain these similarities. One taxon (*Majoranasyriaca*) in particular was found to be among the most quoted species in almost all areas surveyed. CI values, as a measure of traditional botanical knowledge, for edible species in relatively remote and isolated areas (Qalqilia, and Salfit) were generally higher than for the same species in other areas. This can be attributed to the fact that local knowledge of wild edible plants and plant gathering are more spread in remote or isolated areas.

**Conclusion:**

Gathering, processing and consuming wild edible plants are still practiced in all the studied Palestinian areas. About 26 % (26/100) of the recorded wild botanicals including the most quoted and with highest mCI values, are currently gathered and utilized in all the areas, demonstrating that there are ethnobotanical contact points among the various Palestinian regions. The habit of using wild edible plants is still alive in the PA, but is disappearing. Therefore, the recording, preserving, and infusing of this knowledge to future generations is pressing and fundamental.

## Background

Of the Earth's half million plant species, only about 3,000 species have been used as agricultural crops and only 150 species have been cultivated on a large scale. However, while development of genetically modified crops may play an important role in achieving enhanced productivity that is essential for human survival, developing new crops by domesticating currently wild edible species offers considerable potential [1,2].

Millions of people in many developing countries depend on wild resources including wild edible plants to meet their food needs especially in periods of food crisis [3,4]. Many wild edible plants are nutritionally rich [5] and can supplement nutritional requirements, especially vitamins and micronutrients.

Wild edible plants have always been important in the folk traditions of the Mediterranean region [6]. However, food and medicinal uses of these plants have been two of the most relevant and consistent reasons for popular plant management, even in cultures that are increasingly losing their close relationship with nature. It is for this reason that ethno-directed research is very useful in the discovery and development of new drug and food resources [7,8]. It is of outmost importance to obtain data about popular uses of wild edible plants before this knowledge disappears. In many Mediterranean countries these traditions are at risk of disappearing, and hence the crucial need to study such knowledge systems and find innovative ways of infusing them to the future generations [6,9].

During the past decade, several studies have systematically analyzed the consumption and gathering of wild edible plants in specific countries in the Mediterranean area including, e.g.: Greece [10], France [11], Italy and Spain [12-16,9], Turkey [17], Cyprus [18], or in all the Mediterranean area [19].

During the years 2003–2006, a circum-Mediterranean ethnobotanical field survey for wild edible plants was conducted in selected sites in seven countries (Albania, Cyprus, Egypt, Greece, Italy, Morocco, and Spain) [6]. The study has showed that quantity and quality of traditional knowledge varies among the several study countries and is closely related to the traditions, environment and cultural heritage of each country.

In Palestine (West Bank and Gaza), about 2780 plant taxa were recorded as native or naturalized. From the native taxa, 162 taxa were recorded as endemics [20]. The country's diverse topography has permitted the survival of traditional knowledge related to vegetable resources used by locals as food. However, only a few ethnobotanical studies on medicinal plants have been undertaken in some parts of the country [21-24] and no or very little emphasis has been paid to wild edible plants [25,26].

Given the dramatic loss of traditional knowledge regarding wild edible plants, our aim was to document and evaluate the indigenous knowledge, diversity and cultural significance of these plant species in five rural areas of the northern West Bank, comparing the cultural importance of edible plants historically gathered as food and the socio-economic and anthropological context in which these plants have been gathered and processed.

## Methods

### Study sites

**Figure 1:**
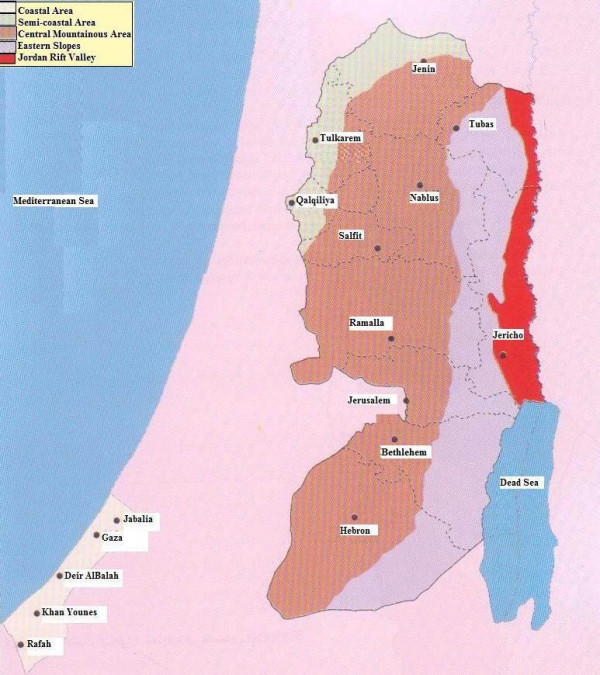
Study areas in the Northern West Bank [31].

Fifteen small communities distributed in five districts in the northern West Bank in the Palestinian Authority (PA) were selected for this study (Table 1 and Figure 1). Each of the five districts was represented by one or more villages (communities) located mainly within homogenous mountainous, rural areas. The environment is mainly semi-arid, and the prevailing socio-economy is agropastoral. Despite its small geographical area, the West Bank is characterized by a large variation in topography (Figure 1). This variation is directly reflected on climate and the distribution and diversification of agricultural patterns, from irrigated agriculture in the Jordan Valley (the lowest area in the world) to rain-fed farming in the mountains [20]. The West Bank is divided into four major biogeographical zones: semi-coastal zone, central highlands, eastern slopes, and the Jordan Rift Valley. The study sites were mainly located in the first three of the above-mentioned zones. The Semi-coastal and central highlands zones represent more than 60 per cent of total area of the West Bank. All rain-fed and nearly half of irrigated land are within this zone. About 105, 000 hectares of fruit trees, namely olives, grapes, almonds, and other deciduous fruit trees are planted in this zone. Most of the winter crops, all summer crops and rain-fed vegetables are grown in this zone. Also, most sheep and goats flocks, cows herds and nearly all poultry farms are located in this zone. The Eastern Slopes zone represents the semi-desert climate as transitional zone between the true Mediterranean and desert climate. It is located between the Jordan Valley and the Central Highland Regions. The steep mountains with little rainfall that predominate in this region make it an almost semi-arid to desert zone. It is suitable for grazing and, to a certain extent, is utilized for field crops varieties that survive with the average yearly rainfall of 150–300 mm such as barley and wheat. Approximately 53.3% of the total population in the West Bank lives in rural areas and refugee camps and the remaining 46.7 of the population in urban areas. The Palestinian per capita GDP averages around US $1,500 annually, mostly coming from service and day labor sector, with agriculture making up between 18 and 30 percent and industry making up very little. Ecological and economic characteristics of the studied localities are outlined in Table 1.

**Table 1:** Number of informants, localities visited and geographical features of each area.

Community/village code	Community/village name	Region/district	Ecological and economic characteristics of the community area	Number of informants
N1	Nablus	Nablus	Mountainous Mediterranean: small scale agriculture, minor industrial activities, tourism nearby.	20
N2	Til	Nablus	Mountainous/rural area: agriculture (olive trees, fig orchards), cattle farms.	20
N3	Hiwara	Nablus	Rural: agriculture (olive trees), minor industrial activities.	10
N4	Yitma	Nablus	Rural: agriculture (olive trees)	10
N5	Qabalan	Nablus	Rural: agriculture (olive trees)	10
N6	Sabastia	Nablus	Rural area: small-scale agriculture, olive trees, tourism.	10
Sub-total				80
J1	Qabatia	Jenin	Rural, internal plane: intensive protected agriculture, olive trees.	10
J2	Fandaqomia	Jenin	Rural area: agriculture (olive trees, stone fruit trees).	10
Subtotal				20
Q1	Azzoun	Qalqilia	Rural area: olive trees.	10
Q2	Kafrthulth	Qalqilia	Rural area: olive trees.	20
Q3	Nabilias	Qalqilia	Rural, semi coastal area: agriculture (olive trees, citrus orchards, intensive agriculture)	10
Subtotal				40
S1	Salfit	Salfit	Mountainous area: agriculture (olive trees).	10
S2	Rafat	Salfit	Rural area: agriculture (olive trees).	10
Subtotal				20
T1	Beitleed	Tulkarm	Rural area: agriculture (olive trees).	20
T2	Baqasharqia	Tulkarm	Rural semi-coastal area: agriculture (olive trees, citrus orchards, intensive agriculture).	10
Subtotal				30
Total				190

### Ethnobotanical data collection and analysis

Interviews were conducted during spring and summer of 2006, with approximately 20–80 informants in each district (total number of interviewees: 190, 138 females, 52 males) (Table 1). They were mainly selected using snowball techniques [27]. Informants with a sound traditional knowledge of useful wild plants, mostly either native born or had been living in the region for more than 30 years, were interviewed. Informants were between the ages of 30 and 95 years with an average age of 52 years.

A clear expression of consent was also obtained before each interview. Through this field study, the ethical guidelines adopted by the International Society of Ethnobiology [28] were observed.

Questions addressed to the informants about wild food consumption were mainly focused on common local name, knowledge about past and present use, mode of consumption and preparation, parts of the plants used, the manner of their preparation and administration, procurement method, place of collection and habitats, threats and conservation status, date/season of collection, method of storage, and period of storage.

Voucher specimens were deposited at the herbarium of Biodiversity and Environmental Research Center at Til, Nablus (West Bank, PA). Identification was carried out using the Flora Palaestina [29] and other available pictorial floras and taxonomic references [30-33].

#### Food use categories of wild edible plants based on folk perceptions

For this study, data were grouped into the following wild edible plants use categories based on folk perceptions: vegetable, fruit, herbal tea, food decoration (used to garnish or ornament food), seasoning, and food preservation. Every plant species mentioned by an informant within one-use category was counted as a one-use-report (UR) [34]. In this work, the term "wild" refers to non-cultivated plants gathered in the field. However, certain consumed species derive from both wild and cultivated specimens, but in such cases all use-reports were considered regardless of the origin of the specimens [34].

#### Estimation of cultural significance of each species (cultural importance index, CI)

The Cultural Significance Index (CI) of each species was estimated for each locality as the summation of the UR in every use-category mentioned for a species in the locality divided by the total number of survey participants (N) in that locality [34]. The additive index takes into account the spread of use (number of informants) for each species and its diversity of food uses. The theoretical maximum value of the index is the total number of different food use categories.

A mean cultural importance index (*mCI*) was used [34] to evaluate wild food plant uses in the Northern West Bank as a whole. It was calculated by considering all localities under study.

#### Estimation of cultural importance of families, CIf

To measure the cultural importance of plant families (CIf), the CIs of the species from each family were added [35].

#### Threats to wild edible plants

To understand local peoples' perception on activities threatening wild edible plants, a number of threatening factors were identified with the community [4]. The factors were presented to informants to choose from. Then the scores from each respondent summed up, the ranks determined and the factor that received the highest total score ranked first.

## Results and Discussion

Additional file 1 presents the plant part used, consumption procedure, food use-category, and number of informants mentioning each use for the 76 wild edible species reported by three informants or above in the five areas.

### Taxonomic diversity

The flora of the study area provides diverse useful species. A total of 100 wild edible plant species are gathered and consumed in the study areas. These are classified among 89 genera and 35 families (Table 2). Seventy six of these plants were mentioned by 3 informants and above and were distributed across 68 genera and 26 families (Additional file 1). Plants that were mentioned by less than 3 informants (24 species, 24 %) were excluded from further discussion (Additional file 2).

**Table 2:** Number of species cited in the study areas by three informants or above, their genera, and families (total numbers of taxa recorded).

	**Nablus**	**Jenin**	**Qalqilia**	**Salfit**	**Tulkarm**	**Total**
No. of Families	21 (25)	22 (23)	24 (30)	24 (27)	16	26 (35)
No. of Genera	60 (68)	50 (53)	59 (69)	44 (48)	35	68 (89)
No. of Species	68 (77)	51 (54)	63 (73)	47 (50)	36	76 (100)

### Plant parts used and modes of consumption

**Figure 2:**
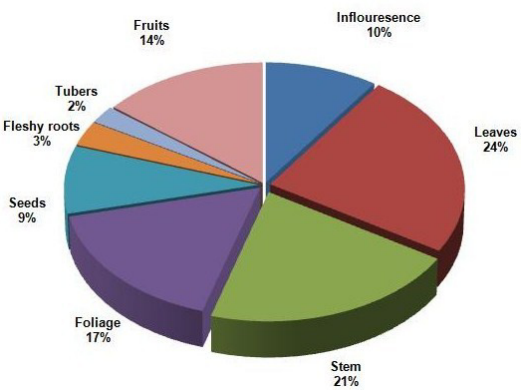
Plant parts used.

Within the edible plants, leaves (24 %), and stems (21 %) were the plant parts most widely used (Figure 2).

The wild edible plants are consumed in many different ways and are prepared using diverse recipes according to local traditions. Some of them are eaten raw, and some others eaten cooked and thus require a more or less complex preparation process (Additional file 1). It is obvious that raw recipes predominate in the modes of consumption with a total percentage of 89 % in the five surveyed areas, while cooked edibles follow with the relatively high percentage of 77 %. This is in agreement with what people think about Mediterranean diets and that Mediterranean people always portrayed eating vegetables raw [36]. However, the high percentage of cooked edibles may be attributed to the change of the socio-economic context of rural areas around the country. Nowadays, people do not spend as much time outside in the natural environment as they used to do in the past in order to feed with raw vegetables, and therefore, since they are influenced by contemporary dietary trends of cooked food, edible plants collected from the wild are brought home for more elaborated cooking recipes.

#### Plants consumed cooked

Many of the wild edible plants (77 %) have been eaten cooked. Some plants are consumed fried in olive oil (e.g., *Rumex acetosa*, *Malva sylvestris*, *Cichorium pumilum*) and especially in an omelette (e.g., *Gundelia tournefortii*).

In some plants, e.g., *Arum palaestinum*, the leaves are cut, boiled and the water is decanted a few times to remove toxic substances, and then the leaves parts are fried using olive oil, and are garnished with lemon.

A number of wild edible plants are used in traditional recipes. For example, the leaves of *Rumex acetosa *are used as filling for a traditional pie called '*sambosek*'. The leaves of *Cyclamen persicum *and *Salvia hierosolymitana *are also used to make 'Za'matoot', and 'Lessaineh', respectively, in which the boiled leaves are filled with rice, minced meat, and condiments and made into rolls before cooked and eaten with yogurt. The inflorescence and leaves of *Gundelia tournefortii *are used to make 'Akoob' in which the inflorescence, young stems and leaves are cut, fried in olive oil, then boiled with meat chops until well done, and then a boiled yogurt suspension is added and the mixture is left to boil for a few minutes before the meal is ready for serving. *Majorana syriaca *is used for preparing a traditional recipe that is very popular in all Palestinian communities called 'za'tar' [37]. The leaves are dried, grinded, mixed with olive oil, sesame seeds, and several other condiments and spices. The mix is then eaten with olive oil and bread.

It is worth mentioning that these recipes have been prepared in similar ways in the different surveyed areas in the present study and also in the Bethlehem area in the southern West Bank of the PA more than 75 years ago [25].

#### Plants consumed raw

Within the five studied localities, most plants (89 %) with edible leaves, roots, or fruits are eaten raw. The majority of these plants are eaten fresh, directly after they are gathered. Many of them (e.g., *Majorana syriaca, Eruca sativa, Foeniculum vulgaris, Portulaca oleracea*) are used in salads and dressed with olive oil and lemon or are eaten with pickled olives, onions and bread. This is in contrast with Hadjichambis et al. [6] findings regarding plants consumed cooked in several Mediterranean countries. On the other hand, many edible fruits are consumed as desserts (*Ceratonia siliqua, Pyrus syriaca, Crataegus aronia, Ziziphus spina-christi*).

#### Preserved plants

A number of plants (49 %) are gathered and preserved to be stored and consumed on longer periods of the year (sometimes all year round, e.g., *Gundelia tournefortii*, *Majorana syriaca*, *Salvia fruticosa*). The most common ways of preserving plants include air drying and then storing in suitable containers (e.g., glass containers), refrigeration, and freezing.

### Most cited plants

Based on number of informants who mentioned the plant for food purposes at different localities (Additional file 1), the following were the most utilized plants (cited by more than half of the maximum number of informants who reported a plant for any food use) (11 species): *Majorana syriaca, Salvia fruticosa, Malva sylvestris, Cyclamen persicum*, *Gundelia tournefortii, Foeniculum vulgare, Arum palaestinum, Rumex acetosa, Matricaria aurea, Micromeria fruticosa*, and *Trigonella foenum-graecum*. These plants have been traditionally used in the five areas.

When the five plants most times quoted in each of the seven Mediterranean countries studied by Hadjichambis et al. [6] are compared with the five plants most quoted in the Palestinian Authority in this study (Table 3) it became clear that within the eight countries the wild edible plants most times quoted show great variability. Again, in the PA, a different group of five species is the most popular, showing only a few similarities with other countries as in the case of *Malva sylvestris *(two countries). According to that data we agree with Hadjichambis et al. [6] suggestion that there is no common Mediterranean cultural heritage in the selected countries regarding the gathered wild edible plants, since even though the most quoted taxa are sometimes the same, the cultural importance of these species is very different in the cuisine.

**Table 3:** Comparison of the five plants most often quoted (scientific name (number of citations)) in each of eight Mediterranean countries.

Area	First plant	Second plant	Third plant	Fourth plant	Fifth plant
Palestine*	*Majorana syriaca *(L.) Rafin. (150)	*Salvia fruticosa *Mill. (131)	*Malva sylvestris *L. (129)	*Cyclamen persicum *Miller (99)	*Gundelia tournefortii *L. (99)
Albania♣	*Chenopodium bonus-henricus *L. (40)	*Fragaria vesca *L. (36)	*Rubus idaeus *L. (35 citations)	*Allium triquetrum *L. (23)	*Rumex alpinus *L. (15)
Greece♣	*Scandix pecten-veneris *L. subsp. pecten-veneris (53)	*Prasium majus *L. (36)	*Sonchus oleraceus *L. (34)	*Cichorium spinosum *L. (32)	*Papaver rhoeas *L. (32)
Cyprus♣	*Silene vulgaris *(Moench) Garcke (17)	*Capparis spinosa *L. (16)	*Asparagus acutifolius *L. (15)	*Malva parviflora *L. (14)	*Scolymus hispanicus *L. (13)
Egypt♣	*Portulaca oleracea *L. (29)	*Beta vulgaris *L. var. cicla L. (24)	*Ziziphus spina-Christi *(L.) Willd (22)	*Corchorus olitorius *L. (20)	*Malva sylvestris *L. (20)
Italy♣	*Cichorium intybus *L. (50)	*Taraxacum officinale *Weber (47)	*Urtica dioica *L. (47)	*Hypochaeris radicata *L. (44)	*Picris echioides *L. (44)
Morocco♣	*Origanium majorana *L. (37)	*Mentha pulegium *L. (31)	*Calamintha officinalis *Moench (27)	*Ficus carica *L.	*Portulaca oleracea *L.
Spain♣	*Tamus communis *L. (15)	*Thymus mastichina *L. (14)	*Andryala integrifolia *L. (15)	*Origanum virens *Hoffmanns. And Link (12)	*Tolpis barbata *(L.) Gaertner (9)

* Present study, ♣ Hadjichambis et al. 2007.

### Species' cultural importance

**Figure 3:**
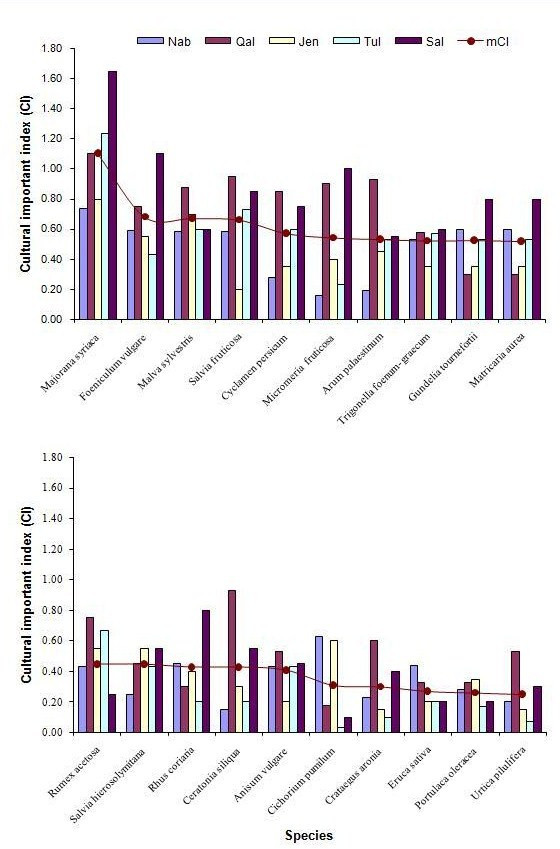
Cultural importance index (CI) of the 20 most relevant species in the study are in descending order by mean value (mCI).

Figure 3 lists, in order of importance, the twenty most culturally important species in the Northern West Bank according to the mCI, and their CI values in each survey area. It is clear that within the five study localities the plant species with highest CI values show large similarity.

As it is expected, these plants include some of the most deeply rooted plants in the Palestinian traditional culture and ethnobotany, namely *Majorana syriaca *(*Za'tar*), *Salvia fruticosa *(*Mariamieh*), *Malva sylvestris *(*Khubbaizeh*), *Cyclamen persicum *(*Za'matoot*), and *Gundelia tournefortii *(*Akoob*). All these plants are used as food and medicine (Additional file 1). Some of these plants are considered holy plants being mentioned in the holy books (e.g., *Majorana syriaca *in the bible), or *sacred*/*blessed *being mentioned in legends linked with holy people (e.g., *Salvia fruticosa *and Virgin Mary; the plant is even called *Mariamieh after *her). *Salvia fruticosa *was also recognized by Palestinian Muslims in Northern Israel for its ritual importance in cemeteries and funerals [38]. *Majorana syriaca*, *Cyclamen persicum*, and *Gundelia tournefortii *have been used for a very long time to prepare traditional recipes [25]. A few of these plants are even mentioned in local folkloric songs and proverbs (*Malva sylvestris, Gundelia tournefortii, Salvia fruticosa*) [25].

The 20 most significant species (Figure 3) include fruits (*Crataegus aronia, Ceratonia siliqua*), seasonings (*Rhus coriaria*, *Foeniculum vulgare*), herbal teas which are used in general as a digestive (*Salvia fruticosa, Matricaria aurea, Anisum vulgare, Trigonella foenum-graecum*), and vegetables (*Malvasylvestris*, *Cyclamen persicum, Arum palaestinum, Gundelia tournefortii, Salvia hierosolymitana, Cichorium pumilum, Rumex acetosa*). For the five areas as a whole, the species used as vegetables were very important, with 4 species in the top 10. Two species used for seasoning and four for herbal tea also rank highly. Fruits were clearly much less important, *Ceratonia siliqua *ranking fifteenth and *Crataegus aronia *ranking eighteenth. Other taxa were less important (Additional file 1).

As it is expected, indigenous people do not eat all wild edible plants present in their environment but only a small part of the local flora. What makes the difference is the cultural decision that is behind each group of gathered food plants [19].

Table 4 also shows the number and percentages of species (% of spp.) and of use report (UR) among each food category at each survey site, which indicate that vegetables were the most important category in all areas (% of spp. = 69.7; % UR = 55.4), followed by herbal teas (35.5; 16.6), plants used for seasoning (26.3; 13.7), and fruits (21.05; 11.2).

**Table 4:** Number and percentage of wild edible plants and of use report (UR) among food-categories at the survey sites.

Number of species (Nsp)												
Food category	N*		Q		J		T		S		Total	
Vegetables	44	65%	44	69%	32	62%	21	58%	30	64%	53	69.74%
Herbal tea	21	31%	13	20%	10	19%	12	33%	12	26%	27	35.53%
Seasoning	15	22%	11	17%	10	19%	9	25%	13	28%	20	26.32%
Fruits	13	19%	12	19%	10	19%	6	17%	7	15%	16	21.05%
Food decoration	4	6%	2	3%	5	10%	2	6%	1	2%	8	10.53%
Food preservation	2	3%	0	0%	0	0%	0	0%	0	0%	2	2.63%
Total	68	146%	64	128%	52	129%	36	139%	47	134%	76	165.79%

Number of use reports (NUR)												

Food category	N		Q		J		T		S		Total	

Vegetables	561	53%	501	65%	171	66%	148	54%	165	48%	1588	55.4%
Herbal tea	249	23%	78	10%	21	8%	65	24%	53	15%	476	16.6%
Seasoning	125	12%	72	9%	31	12%	39	14%	83	24%	394	13.7%
Fruits	114	11%	117	15%	27	10%	19	7%	40	12%	321	11.2%
Food decoration	9	1%	2	0%	8	3%	4	1%	4	1%	81	2.8%
Food preservation	9	1%	0	0%	0	0%	0	0%	0	0%	9	0.3%
Total	1067	100%	770	100%	258	100%	275	100%	345	100%	2869	100%

*N, Nablus; Q, Qalqilia; J, Jenin; T, Tulkarm; S, Salfit.

### Differences in CI values for species among the different areas

Figure 3 indicates cultural importance index (CI) of the 20 most relevant species in the study are in descending order by mean value (mCI).

Figure 3 also shows appreciable differences among the CI values of the 20 most relevant species obtained in the different areas. All the ten species with the highest mCI, were cited in all five areas. Moreover, most were important in every region. The next ten species were also used at all study sites, e.g., *Matricaria aurea, Rumex acetosa*. A common cultural background may explain these similarities. Interestingly, the CI values for edible species (9/20) in Qalqilia, and Salfit (5/20) are generally higher than for species in other areas (Figure 3). This can be attributed to the fact that local knowledge of wild edible plants and plant gathering are more spread in remote or isolated areas [34]. To examine this supposition further, the CI values for all species in each area (mCIa) as a measure of botanical knowledge were calculated. The mCIa value for Salfit (0.36) was more than that of Tulkarm (0.26). This is an appreciable difference in knowledge of edible plants among different human groups. Qalqilia, a neighboring territory had also a comparably high mCIa value (0.31), followed by Jenin (0.25), and Nablus (0.19). Although Tulkarm and Qalqilia (both semi coastal areas) are neighboring territories sharing a similar environment, the difference is significant. This can be explained by greater loss of knowledge in the former. Moreover, Qalqilia and Salfit were partly isolated either due to movement restrictions being located in an area of extensive Israeli settlement activities, in case of Salfit, or isolated by Israeli separation/security wall in case of Qalqilia.

### Cultural Importance of the families

**Figure 4:**
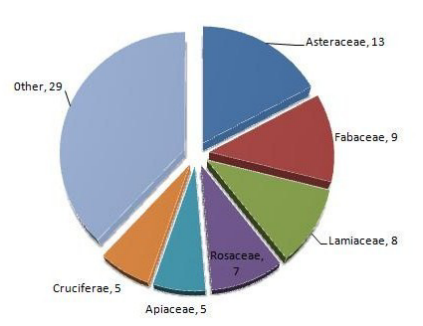
Most quoted wild food botanical families in the study areas.

A comparison between the most quoted food botanical families in the different communities is presented in Figure 4. The data were expressed as percentage of the wild food taxa belonging to a given family out of the total number of quoted taxa recorded in the related area. Asteraceae (13 species, 17%), Fabaceae (9, 12 %), and Lamiaceae (8, 11 %) were more often quoted as wild edible plants in all localities than were other families, followed by Rosaceae (7, 9 %), and Brassicaceae and Apiaceae (5, 5 %). The last three families were more quoted in Nablus than in other localities. Other families showed no or low representation (0–3 spp.) in the different localities (Table 5). Our results are therefore in agreement with those of Hadjichambis et al [6], who also found Asteraceae, Lamiaceae, Rosaceae, and Apiaceae to be among the five families with the greater number of representative wild edible plants in the Mediterranean countries they studied. These are known to be big families with many representatives in the Mediterranean area and some of which are common plants [6]. Hence our results confirm that people tend to use preferably the plants that are easily available to them. These results are in agreement with those of Bonet and Valle's [15], Bonet et al. [39], Johns et al. [40], and Stepp and Moerman [41], which affirmed that the more common a plant (family or species) is in an area, the greater is the probability of its popular use.

**Table 5:** Cultural importance of some of the most important families in each of the surveyed areas, in descending order of the mean estimated for the whole North West Bank (mCIf).

Family	N*	Q	J	T	S	mCIf
	2.375	3.7	2.65	2.77		
Lamiaceae (Labiatae)	3.39	4.78	3.35	3.56	4.55	3.21
Asteraceae (Compositae)	2.03	2.46	1.90	0.87	1.85	1.82
Fabaceae (Leguminosae, Papilionaceae)	1.17	2.53	1.25	1.03	1.40	1.48
Apiaceae (Umbelliferae)	1.03	1.60	0.80	0.90	1.55	1.18
Malvaceae	0.63	0.88	0.70	0.60	0.60	0.68
Polygonaceae	0.61	0.95	1.10	0.27	0.30	0.65
Araceae	0.23	1.00	0.45	0.53	0.85	0.61
Rosaceae	0.78	0.80	0.70	0.20	0.45	0.59
Primulaceae	0.30	0.85	0.35	0.60	0.80	0.58
Brassicaceae (Cruciferae)	.89	0.30	0.75	0.30	0.20	0.49
Anacardiaceae	0.40	0.83	0.45	0.20	0.55	0.49
Portulacaceae	0.28	0.33	0.35	0.15	0.20	0.26
Urticaceae	0.20	0.53	0.15	0.07	0.30	0.25
Liliaceae	0.68	0.13	0.10	0.03	0.15	0.22
Lauraceae	0.26	0.33	0.05	0.10	0.10	0.17
Fagaceae		0.43	0.20	0.03	0.10	0.15
Iridaceae	0.21	0.18	0.05		0.15	0.12
Myrtaceae	0.15	0.08			0.35	0.12
Rutaceae	0.038	0.15	0.05			0.05
Rhamnaceae	0.01	0.33	0.05	0.00	0.05	0.09
Boraginaceae	0.01	0.08	0.15		0.15	0.08
Oxalidaceae		0.30			0.05	0.07
Caryophyllaceae	0.05	0.05			0.20	0.06
Solanaceae	0.04	0.03	0.15			0.04
Capparidaceae			0.10		0.10	0.04
Geraniaceae	0.03				0.10	0.03

*N, Nablus; Q, Qalqilia; J, Jenin; T, Tulkarm; S, Salfit.

**Figure 5:**
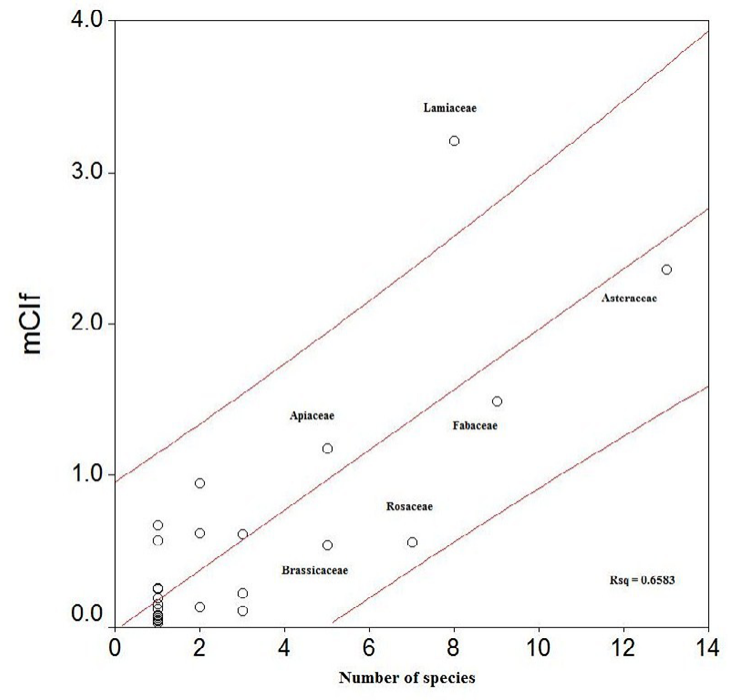
**Regression of the cultural importance of the families (mCIf) on the number of species in the family.** Discontinuous line marks the 95% confidence interval.

Although a family's cultural importance correlates strongly (r = 0.615) with the number of species in each family (Figure 5) a regression analysis was carried out to confirm statistically which families had higher values than expected for the number of species [34]. Figure 5 indicates that the plant families with more than 5 species and greater cultural importance as wild food in the Northern West Bank were: Lamiaceae, Asteraceae, Fabaceae, Apiaceae, Rosaceae and Cruciferae. However, only the Lamiaceae attains a significantly higher figure for cultural importance than that expected for the number of species. This fact remarks the high significance of vegetable and herbal tea category in most of the survey areas.

A local particularity that is worth mentioning is that Lamiaceae is the most important family in Salfit, according to its CIf (3.21, see Table 5). This is due to the higher relevance of plants used as herbal tea in this area, both in relative number of species % of spp. and UR, as shown in Table 4 and Additional file 1.

### Contribution of wild edible plants as food medicines

From the 103 edible plants recorded, 64 (62 %) have been cited to be used for food as well as for medicine (Additional files 1 and 2). These are food plants that receive recognition as medicinal in the Traditional Arabic Palestinian Herbal Medicine and represent a part of the Palestinian medicinal ethnoflora [24]. Overlapping between food and medicine is well known in traditional societies [9] and represent an often neglected field in ethnopharmaceutical research [6].

No clear dividing line between food and medicinal plants usually exists, especially in indigenous and local traditions. Food can be used as medicine and vice versa. Still certain wild edible plants are used because of their assumed health benefits and thus can be called medicinal foods [42].

For example, the leaves of *Arum palaestinum *is consumed fried using olive oil because it is perceived to protect from colon cancer.

The relatively common use of Lamiaceae and Asteraceae as food as well as medicine in the present study is in agreement with the findings of a similar study on food plant consumption in seven Mediterranean countries [6]. This can be attributed to the phytochemical features of many species of these families (i.e., presence of essential oils in Lamiaceae, and sesquiterpene lactones in Asteraceae).

It is worth mentioning that previous ethnobotanical field surveys [18,24] have revealed that healthcare practices of the household using preparations based on plants are usually administered by women. However, most studies have advantaged the 'medicine of healers' instead of the 'medicine of the households' [43,24].

### Threats and conservation status

The study revealed that 42 % of the wild edible plant species were gathered from natural shrub lands, followed by agricultural fields (35 %), roadsides (14 %), and natural forests (9 %). Wild edible plants are facing threats in their natural habitats from various human activities. The level of impacts of these activities varied from place to place. The local people's perception on the activities more threatening to wild edible plant species (over-grazing, agricultural land expansion, over-harvesting, uncontrolled fire setting and fuel wood collection) for each factor (total some of each factor), varied among informants of different communities (Table 6).

**Table 6:** Results of the ranking of factors considered as threats to wild edible plants.

Factors	Respondents															Total	Rank
			
	*N1	N2	N3	N4	N5	N6	J1	J2	S1	S2	T1	T2	Q1	Q2	Q3		
Agricultural land expansion	9	17	7	3	10	6	7	7	9	3	16	6	5	5	10	120	1
Over- grazing	9	11	9	6	10	3	5	6	1	5	14	7	10	9	1	106	2
Over-harvesting	4	4	0	5	10	1	4	1	4	4	4	2	1	3	6	53	3
Uncontrolled Fire setting	10	6	1	3	0	2	3	0	1	0	5	3	7	2	0	43	4
Fuel wood collection	1	1	0	2	1	0	5	0	0	0	8	0	2	0	0	20	5

* N = Nablus; J = Jenin; S = Salfit; T = Tulkarm; Q = Qalqilia. The values in the Table are the number of informants who mentioned the factor at the different sites from each community.

However, the overall rating for all communities showed agricultural expansion as the main threat to wild plant species, followed by over grazing and over-harvesting. As to the protection status, most of the wild species in these areas have no protection. However, very few economic plant species (e.g., *Majorana syriaca*) are now cultivated and marketed by some farmers. This shows that acquisition of economic benefits from species might promote local people's interest in conservation and maintenance of such locally important and threatened species [4].

## Conclusion

The data we have presented here showed that gathering, processing and consuming wild edible plants are still important activities in all the selected areas.

Many wild edible plants have been quoted and cited in the different areas, demonstrating that there is a common cultural heritage in these areas regarding the gathered food plants, since most quoted taxa are the same and the cultural importance of these taxa is very similar in the local cuisine. However, a few differences in the gathering, processing and consumption of wild edible plants between these areas were observed. In the Salfit and Qalqilia areas, the mean cultural importance values of wild edible botanicals were considerably higher than those in the other areas. The differences may be due to the likelihood that in Salfit and Qalqilia the erosion of traditional knowledge on wild edible plants is taking place more slowly.

The consumption of wild edible plants is an addition or a complement to a diet of cultivated food plants, while the quantity and quality of traditional knowledge varies slightly among the studied localities. The similar culture, religious, beliefs, ecologic backgrounds and historic development around the different localities of the PA resulted in similar diets that resolve around the local traditions.

In the PA, fewer wild edible plants are being used at present time than in previous decades. It is clear that with change in nutritional habits and the influence of contemporary western life style, younger generation has lost the traditional knowledge necessary to identify, gather and process these species.

The decline in wild food gathering appears to be due to several factors including socio-economic conditions (life style, improvement in national road network); agricultural practices (i.e., spread of intensive agriculture), and thus easier access to and higher availability of agricultural products, and negative connections (i.e., many middle-aged people perceive the consumption of wild edible plants in a negative way as a symbol of poverty of the past).

The habit of using wild edible plants is still alive in the PA, but is 'aeging'. Therefore, the recording and preserving of this knowledge is pressing and fundamental. Such TK can be disseminated to future generations through developing novel curricula and instructive material in schools and universities [6,44],

For the five areas as a whole, the species used as vegetables were the most important category (68.4% of use-reports), followed by herbal teas (35.4%), plants used for seasoning (26.6 %), and fruits (21.5 %). The results show that culturally most important families in descending order of mCIf were Lamiaceae, followed by Asteraceae, Fabaceae, and Apiaceae. Lamiaceae species are very important as herbal teas and seasoning, Asteraceae, Fabaceae, and Apiaceae as vegetables,

As to the protection status, most of the wild species in the surveyed areas have no protection. However, very few plant species (e.g., *Majorana syriaca*) are now cultivated by some farmers (an area of about 106 hectares were planted with thyme in the West Bank in 2005 with an annual yield of 1948 tons [45]), thus reducing threats to endangered wild edible plants and their habitats. This shows that acquisition of economic benefits from species might promote local people's interest in conservation and maintenance of such locally important and threatened species. Sustaining wild edible plants is meaningful only if conservation efforts take into account the food plants inextricable connections with cultural heritage.

## Authors' contributions

JHA–S, WAE, FAK, KHQ, ISK, IMS, AAM, BAI, HMH, RBK, SMA, GMS, MAA, MMH–A, NAS, HKA, and HAN collected the data in 15 villages from the six study areas and contributed to the discussion. MSA-S and RMJ analyzed the data and drafted the theoretical framework for the discussion.

## Supplementary Material

Additional file 1 Wild food plants (cited by ≥ 3 informants) traditionally consumed and number of informants that mentioned each food- use in the survey areas. Click here for file

Additional file 2 Wild food plants (cited by 1 or 2 informants) traditionally consumed and number of informants that mentioned each food-use in the survey areas. Click here for file
