# Supplementary material for: Traditional knowledge of wild edible plants used in Palestine (Northern West Bank): A comparative study
Source: J Ethnobiol Ethnomed. 2008 May 12;4:13. doi: 10.1186/1746-4269-4-13 (PMC2396604; doi:10.1186/1746-4269-4-13)
Supplement: Additional file 1 — Wild food plants (cited by ≥ 3 informants) traditionally consumed and number of informants that mentioned each food- use in the survey areas. [file 1746-4269-4-13-S1.pdf]

Additional file 1. Wild food plants (cited by ≥ 3 informants) traditionally consumed and number of informants that mentioned each food- use in the survey areas. N, Nablus; Q, Qalqilia; J, Jenin; T, Tulkarm; S, Salfit.

Latin name	Arabic common name	No. of informants	N	Q	J	T	S	Food use category	Part used, way of consumption
**Anacardiaceae**									
Pistacia lentiscus L. *	*Sarrees*	25	9	5			2	Fruits	Seeds, eaten raw or roasted
				12	1			Seasoning	Lentisk, added to cake as a condiment
Pistacia palaestina Boiss. *	*Butum Falastini*	10		8			2	Fruits	Fruits, stewed and eaten
							2	Seasoning	Buds, added to thyme as a condiment
Rhus coriaria L. *	*Summak*	51	23	11	6	4	7	Seasoning	Fruits, use as condiment on food and thyme
**Apiaceae (Umbeliferae)**									
Ammi visnaga (L.) *	*Kheleh*	3		1				Herbal tea	Seeds, as herbal tea
				2				Vegetables	Inflorescence, and leaves, are added to salty preserved food
Eryngium creticum Lam. *	*Qarsa'neh*	10	1	7	1	1		Vegetables	Leaves and stems, eaten raw as salad with garlic and yoghurt, or cooked as pastry
Daucus carota L. ssp.*maximus* (Desf.) Ball	*Jazar Barri*	3		3				Vegetables	Fleshy roots, eaten raw
Anisum vulgare Gaertn. (*Pimpenella anisum* L.) *	*Yansoon*	61	15	1		6	3	Herbal tea	Seeds, as herbal tea
			18	10	1			Fruits	Seeds, used to make cake and sweets
			1		2	7	6	Seasoning	Seeds, used in cake as a condiment

Foeniculum vulgare Mill. *	*Shomar*	87	18	1				Herbal tea	Seeds, as herbal tea
			2	1	1	2	4	Food decoration	Seeds, boiled and added to bread
			2					Food Preservation	Leaves, dried and added to pickles
			3		3	2	10	Fruits	Foliage, eaten raw
			12	11	5	3	8	Seasoning	Foliage, dried and added to cake as a condiment
			10	17	2	6		Vegetables	Foliage, eaten raw as salad, or cooked as soup
**Araceae**									
Eminium spiculatum (Blume) Kuntze*	*Smaia'*	12	2	4			6	Vegetables	Leaves, raw, added to salad
Arum palaestinum Boiss *	*Lufe*	84	5			2		Herbal tea	Leaves, as herbal tea
			10	37	9	14	11	Vegetables	Leaves, cooked
**Asteraceae (Compositae)**									
Scorzonera papposa DC.	*Makhadet Alarous*	23	10	7			6	Vegetables	Stem and leaves, raw as salad, eaten raw
Silybum marianum (L.) Gaertn. *	*Sinnariah*	13	6	2	4	1		Vegetables	Stem, and seeds, cooked as soup, eaten raw
Tragopogon coelesyriacus Boiss. *	*Thennabeh Faras*	15	5	6			4	Vegetables	Stem, eaten raw
Chrysanthemum coronarium L. *	*Bisbas*	10	1					Herbal tea	Inflorescence, herbal tea
			2					Seasoning	Foliage, added to fish as condiment
			2	3	1	1		Vegetables	Stems, eaten raw
Anthemis palestina Reut. *	*Oqhuwan*	7	3	1	2	1		Herbal tea	Inflorescence, as herbal tea
Carlina hispanica Lam.	*Zind Abd*	7	1	6				Vegetables	Leaves, eaten raw
Onopordum carduiforme Boiss	*Za'keer*	4	3		1			Vegetables	Stems, eaten raw
Centaurea dumulosa Boiss *	*Murrar*	38	11	16	3		8	Vegetables	Stem, eaten cooked, or raw as salad

Cichorium pumilum Jacq . *	*Hendba'*	70	50	6	12	1	1	Vegetables	Leaves, boiled and eaten as salad with yoghurt
Gundelia tournefortii L. *	*Akoob*	99	48	12	7	16	16	Vegetables	Young stems, inflorescence, cooked with meat and yoghurt.
Lactuca saligna L. *	*Khus Barri*	37	10	15	4	2	4	Vegetables	Leaves, eaten raw as salad, or eaten raw
Matricaria aurea (Loefl.) Sch. Bip. *	*Babounej*	83	41	13	3	9	10	Herbal tea	Inflorescence, as herbal tea
			1	2	1	11		Seasoning	Foliage, added to tea as a condiment
Notobasis syriaca (L.) Cass. *	*Khurfaish*	41	16	15	6		4	Vegetables	Stems, eaten raw
**Boraginaceae**									
Anchusa strigosa Banks & Sol.	*Hemhem*	10			3			Herbal tea	Inflorescence, raw as juice
			1	3			3	Vegetables	Leaves, eaten raw as salad
**Brassicaceae (Cruciferae)**									
Sinapis arvensis L. *	*Khardal Barri*	34	1					Seasoning	Seeds, added to food as condiment
			24	1	6	3		Vegetables	Leaves and stem, eaten raw, or added to salad
Lepidium sativum L. *	*Rashad*	16		1	2			Food decoration	Foliage, added to food for decoration
			13		1			Vegetables	Leaves, raw as salad
Diplotaxis erucoides (L.) DC. *	*Hewaireh*	8	6		2			Vegetables	Stem, eaten raw, or added to salad
Raphanus rostratus DC.	*Fijjaileh*	3	3					Seasoning	Foliage, eaten raw
Eruca sativa Miller *	*Jarjeer*	48	25	10	3	6	4	Vegetables	Foliage, eaten raw as salad
**Capparidaceae**									
Capparis spinosa L. *	*Cappar*	3			1		2	Vegetables	Fruits, preserved as pickles
**Caryophyllaceae**									
Paronychia argentea Lam. *	*Rijl Hamameh*	10	3	1			2	Herbal tea	Foliage, herbal tea
			4	2			4	Vegetables	Foliage, cooked as pastry or eaten raw

**Fabaceae (Leguminosae, Papilionaceae)**									
Vicia narbonensis L.	*Burraideh*	19	6	8			5	Vegetables	Seeds, cooked as snack, eaten raw or roasted
Lathyrus blepharicarpus Boiss.	*Saisa'*	31	9	16		3	3	Fruits	Fruits, eaten raw
Trigonella sp. *	*Hellaibeh*	9	9					Fruits	Seeds, fleshy seeds eaten raw
Glycyrrhiza glabra L.	*Irq sous*	5	5					Herbal tea	Leaves and fruits, dried and prepared as juice
Psoralea bituminosa L.	*Owaineh*	9	1	1	7			Vegetables	Fruits, stewed and eaten, or cooked as pastry, eaten raw
Cassia senna L. *	*Sanamokeh*	3		1		2		Herbal tea	Leaves, as herbal tea
Ceratonia siliqua L. *	*Karoob*	53	12	25	4	5	8	Fruits	Fruits, eaten raw, prepared as jam, cooked and eaten with bread
Tetragonolobus palaestinus Boiss. & Blanche	*Saiba'h*	36	10	17	5	4		Vegetables	Fruits, eaten raw
			11	14	5	4		Fruits	Fruits, eaten raw
Trigonella foenum- graecum L. *	*Hilbeh*	75	18			5	7	Herbal tea	Seeds, as herbal tea
			16	16	2	6	5	Fruits	Seeds, cooked to make sweets
			8	7	5	6		Vegetables	Seeds, cooked as snack
**Fagaceae**									
Quercus calliprinos Webb. *	*Sendian*	22		17	2			Herbal tea	Fruits, dried and grounded then added to coffee
					2	1	2	Fruits	Fruits, roasted
**Geraniaceae**									
Erodium gruinum (L.) L'Her	*Ibret Ajouz*	4	2				2	Vegetables	Stems, cooked and eaten as snack, or eaten raw
**Iridaceae**									
Crocus hermoneus subsp. *palaestinus* N. Feinbrun	*Um Qais*	12	8	4				Vegetables	Bulbs, roasted
Crocus hyemalis Boiss. & Blanche *	*Bizazeh*	8	4	3	1			Vegetables	Bulbs, roasted
Crocus sativus L. *	*Za'faran*	5	4				1	Seasoning	Leaves, added for food as a condiment

**Lamiaceae (Labiatae)**									
Teucrium polium L. *	*Jedeh Subian*	27	8		2	1	2	Herbal tea	Leaves, herbal tea
				9	4	1		Vegetables	Leaves, cooked as soup, cooked with egg, wheat, and water) omelet
Coridothymus capitatus (L.) Reichb. *	*Za'tar Farsi*	25	7			2		Herbal tea	Leaves, herbal tea
			1					Seasoning	Leaves, raw added to pickles
			11	3	1		2	Vegetables	Leaves, dried and added to thyme
Origanum majorana L. *	*Mardaqoush*	7	6		1			Herbal tea	Foliage, herbal tea
Majorana syriaca (L.) Rafin. *	*Za'tar Barri*	150	22			6	4	Herbal tea	Leaves, as herbal tea
					2			Food decoration	Leaves, fresh leaves added to food for decoration
			2	7		3	11	Seasoning	Leaves, added to meat as a condiment
			35	35	14	28	18	Vegetables	Leaves, cooked as pastry, dried and mixed with wheat, salt, …
Mentha longifolia L. *	*Na'na' Barri*	25	12				2	Herbal tea	Leaves, as herbal tea
			2		1			Food decoration	Leaves, cooked as pastry
			1				1	Seasoning	Leaves, added to salad
			3		5		3	Vegetables	Leaves, dried and added to thyme
Micromeria fruticosa (L.) Druce. *	*Za'tar Balat*	79	9	3	2	1	5	Herbal tea	Leaves, as herbal tea
						2		Food Decoration	Leaves added to salad
			4	6	6		14	Seasoning	Leaves, added to tea, or used as a condiment
				27		4	1	Vegetables	Leaves, cooked as pastry
Salvia fruticosa Mill. *	*Mariamieh*	131	40	35		19	7	Herbal tea	Foliage, herbal tea
			7	3	10	3	10	Seasoning	Leaves, added to tea
Salvia hierosolymitana Boiss. *	*Lessaineh*	69	18	16	11	13	11	Vegetables	Leaves, eaten cooked with rice and meat

**Lauraceae**									
Laurus nobilis L. *	*Ghar*	40	21	13	1	3	2	Seasoning	Leaves, added to food as a condiment
**Liliaceae**									
Allium ampeloprasum L. *	*Thoum Barri*	37	7					Food preservation	Leaves, added to pickles
			14			1	2	Seasoning	Foliage, used as condiment on food
			17	4				Vegetables	Leaves, eaten raw with bread
Asparagus aphyllus L. *	*Halion*	9	6		2		1	Vegetables	Stems, cooked as a snack
Asphodeline lutea (L.) Reichenb. *	*Otaat*	11	10	1				Vegetables	Stems, raw as snack
**Malvaceae**									
Malva sylvestris L. *	*Khubbaizeh*	129	50	35	14	18	12	Vegetables	Leaves, cooked as a snack, or cooked and eaten with rice
**Myrtaceae**									
Myrtus communis L. *	*Reehan*	15	5				1	Herbal tea	Leaves, added to tea
			3	2			2	Food decoration	Leaves, raw in salads
			4	1			4	Seasoning	Leaves, added to food as condiment
**Oxalidaceae**									
Oxalis pes-caprae L	*Hamsees*	13		12			1	Vegetables	Leaves, cooked as pastry
**Polygonaceae**									
Rumex acetosa L. *	*Hummaid*	84	34	30	11	8	1	Vegetables	Leaves, cooked as pastry, or eaten raw as salad
Rumex patientia L. *	*Salq*	39	15	8	11		5	Vegetables	Leaves, cooked with meat, or prepared as pastry , or added to soup
**Portulacaceae**									
Portulaca oleracea L. *	*Baqleh*	49	22	13	7	3	4	Vegetables	Foliage, cooked or eaten raw as salad

**Primulaceae**									
Cyclamen persicum Miller *	*Za'matoot*	99	24	34	7	18	16	Vegetables	Leaves, cooked with rice and meat, or raw as salad
**Rhamnaceae**									
Ziziphus sativa Gaetn.	*Innab*	5	1	3	1			Fruits	Fruits, eaten raw
Ziziphus spina-christi (L.) Mill. *	*Seder*	10		1				Fruits	Fruits, eaten raw
				9			1	Vegetables	Leaves, cooked and eaten as snack
**Rosaceae**									
Pyrus syriaca Boiss *	*Ajas Barri*	18	13	1	3	1		Fruits	Fruits, eaten raw
Prunus mahaleb L. *(Cerasus mahaleb)* *	*Mahleb*	17		2				Herbal tea	Seeds, herbal tea
			11	2	1	1	1	Seasoning	Seed, added to stored cheese as a condiment
Rubus sanctus Schreb. *	*Ollaiq*	12	11	1				Fruits	Fruits, eaten raw, or prepared as jam
Amygdalus communis L. *	*Louz Hilo*	5	1		2			Food decoration	Seeds, cooked and added to food
			1		3			Fruits	Fruits, eaten raw
					1			Seasoning	Seed, dried added to sweats
					1			Vegetables	Seeds eaten raw
Amygdalus korschinskii Hand.-Mazz	*Louz Barri*	4	1					Food decoration	Seeds, dried seed added at the surface of cooked rice
			1	1				Fruits	Seeds, cooked and added to food
				1				Vegetables	Fruits, preserved in salt solution pickles
Rosa centifolia L.	*Ward Jouri*	3	3					Herbal tea	Inflorescence, boiled and prepared as juice
Crataegus aronia L. Bosc. Ex DC *	*Za'roor*	58	20	24	3	3	8	Fruits	Fruits, eaten raw

**Rutaceae**									
Ruta chalepensis L. *	*Faijen*	10	2		1			Herbal tea	Leaves, herbal tea
			1	6				Vegetables	Foliage, cooked or eaten raw as salad
**Solanaceae**									
Mandragora autumnalis Bertol.	*Tuffah Majan*	7	3	1	3			Fruits	Fruits, eaten raw
**Urticaceae**									
Urtica pilulifera L. *	*Qurrais*	48	15		3	2	6	Herbal tea	Leaves, herbal tea
			1	21				Vegetables	Stem, eaten raw as salad

* Used also as a medicinal plant
