# Supplementary material for: Traditional knowledge of wild edible plants used in Palestine (Northern West Bank): A comparative study
Source: J Ethnobiol Ethnomed. 2008 May 12;4:13. doi: 10.1186/1746-4269-4-13 (PMC2396604; doi:10.1186/1746-4269-4-13)
Supplement: Additional file 2 — Wild food plants (cited by 1 or 2 informants) traditionally consumed and number of informants that mentioned each food-use in the survey areas. [file 1746-4269-4-13-S2.pdf]

Additional file 2. Wild food plants (cited by 1 or 2 informants) traditionally consumed and number of informants that mentioned each food- use in the survey areas.

Latin name	Arabic common name	No. of informants	N	Q	J	T	S	Food use category	Part used, way of consumption
**Apiaceae (Umbeliferae)**									
Ferula communis L.	*Kalkh*	2			2			Vegetables	Inflorescence, eaten cooked
**Brassicaceae (Cruciferae)**									
Capsella bursapastoris (L.) Medik.	*Kees Rai*	1		1				Vegetables	Leaves, eaten raw with bread
**Caryophyllaceae**									
Dianthus strictus Banks & Sol.	*Koronful*	1	1					Herbal tea	Inflorescence, as herbal tea
**Cucurbitaceae**									
Bryonia syriaca Boiss.	*Battikh Barri*	1		1				Fruits	Fruits, eaten raw
**Dioscoraceae**									
Tamus communis L. *	*Jarma'*	2		1				Vegetables	Leaves, cooked with onion and tomato
**Euphobiaceae**									
Euphorbia helioscopa L. *	*Lebbaineh*	2	2					Fruits	Leaves, eaten raw
**Fabaceae (leguminosae, Papilionaceae)**									
Lupinus albus L.	*Turmos*	2	2					Fruits	Seeds, eaten boiled.
Tetragonolobus purpureus Moench	*Jalthone*	1					1	Herbal tea	Leaves, as herbal tea
Scorpiurus muricatus L.	*Anjadeh*	1					1	Vegetables	Leaves, eaten raw
Calycotome villosa (Poir.) Link	*Qundail*	1	1					Food decoration	Inflorescence, added to traditional margarine
**Fagaceae**									
Castanea vulgaris (Lam.)	*Castana'*	1			1			Fruits	Fruits, roasted
**Geraniaceae**									
Erodium spp	*Bukhtrieh*	2		2				Vegetables	Leaves, eaten cooked

**Lamiaceae (Labiatae)**									
Calamintha incana (Sm.) Helder	*Za'tar Sidi Musa*	1	1					Herbal tea	Leaves, as herbal tea
Mellisa officinalis L. *	*Mellisa*	2		2				Seasoning	Leaves, added to tea as a condiment
**Malvaceae**									
Hibiscus sabdariffa L.	*Karkadaih*	2	2					Herbal tea	Leaves, as herbal tea
**Moraceae**									
Ficus sycomorus L.	*Jummaiz*	2		1				Fruits	Fruits eaten raw
**Orchidaceae**									
Orchis mascula (L.) L.	*Sahlab*	1	1					Seasoning	Leaves, dried and added to boiled milk
**Papaveraceae**									
Glaucium arabicum Fresen.	*Bakhit*	1		1				Vegetables	Leaves, eaten cooked
Papaver argemone L.	*Hannoun Araies*	1					1	Vegetables	Leaves, eaten raw
**Poaceae (Gramineae)**									
Saccharum ravennae (L.) Murray	*Qussaib*	1			1			Herbal tea	Leaves, as herbal tea
**Rhamnaceae**									
Rhamnus palaestinus Boiss.	*Suwwaid*	1		1				Fruits	Fruits, eaten raw
**Rosaceae**									
Prunus ursina Kotsch.	*Barqook Barri*	2	1	1				Vegetables	Fruits, eaten raw
Celtis australis L.	*Mays*	1	1					Fruits	Fruits eaten raw
**Zygophyllaceae**									
Nitraria schoberi L. *	*Arqad*	1		1				Vegetables	Foliage, eaten cooked

* Used also as a medicinal plant
